# Hemisphere-specific, differential effects of lateralized, occipital–parietal α- versus γ-tACS on endogenous but not exogenous visual-spatial attention

**DOI:** 10.1038/s41598-020-68992-2

**Published:** 2020-07-23

**Authors:** Florian H. Kasten, Tea Wendeln, Heiko I. Stecher, Christoph S. Herrmann

**Affiliations:** 1 Experimental Psychology Lab, Department of Psychology, Cluster for Excellence “Hearing for All”, European Medical School, Carl Von Ossietzky University, Ammerlaender Heerstr. 114-118, 26129 Oldenburg, Germany; 2 Neuroimaging Unit, European Medical School, Carl Von Ossietzky University, Oldenburg, Germany; 3 Research Center Neurosensory Science, Carl Von Ossietzky University, Oldenburg, Germany

**Keywords:** Attention, Human behaviour

## Abstract

Orienting spatial attention has been associated with interhemispheric asymmetry of power in the α- and γ-band. Specifically, increased α-power has been linked to the inhibition of unattended sensory streams (e.g. the unattended visual field), while increased γ-power is associated with active sensory processing. Here, we aimed to differentially modulate endogenous and exogenous visual-spatial attention using transcranial alternating current stimulation (tACS). In a single-blind, within-subject design, participants performed several blocks of a spatial cueing task comprised of endogenous and exogenous cues while receiving lateralized α- or γ-tACS or no stimulation over left or right occipital-parietal areas. We found a significant, differential effect of α- and γ-tACS on endogenous (top-down) spatial attention but not on exogenous (bottom-up) attention. The effect was specific to tACS applied to the left hemisphere and driven by a modulation of attentional disengagement and re-orientation as measured during invalid trials. Our results indicate a causal role of α-/γ-oscillations for top-down (endogenous) attention. They may further suggest a left hemispheric dominance in controlling interhemispheric α-/γ-power asymmetry. The absence of an effect on exogenous attention may be indicative of a differential role of α-/γ-oscillations during different attention types or spatially distinct attentional subsystems.

## Introduction

At any given moment, our sensory systems are confronted with an overwhelming amount of information. In order to maintain the ability of goal directed behavior, it is necessary to perform attentional selection, i.e. inhibition of irrelevant and facilitation of relevant information. Two distinct types of attention can be distinguished. Bottom-up (exogenous) attention is described as passive, reflexive and involuntary (e.g. caused by a sudden change of visual input in the periphery), whereas top-down (endogenous) attention is active and voluntary^1^.

Spatial attention refers to the ability to focus attention on specific locations e.g. of the visual field or auditory scene. If the location of an upcoming target stimulus is indicated by a preceding stimulus (cue), faster reaction times (RTs) are commonly observed for correctly (validly) cued targets, whereas slower RTs are observed for incorrectly (invalidly) cued targets^2^. In recent years, a growing body of research has linked spatial attention in different sensory domains to inter-hemispheric asymmetry of power in the α- and γ-frequency bands^3–11^. More specifically, α-oscillations are hypothesized to reflect inhibition of task irrelevant cortical areas^12,13^, while γ-oscillations are thought to reflect active information processing^12^. In line with this idea, increased power in the α-frequency band is commonly observed over cortical areas that process inputs from the unattended hemifield^3–7,10,11^, while increased γ-oscillations have been observed over regions responsible for processing the attended locations^8,10,11^. Interestingly, asymmetry in the α-band appears to occur earlier (after the cue) than in the γ-band, which is strongest after onset of the target stimulus^10^. Further, alterations of α-power lateralization have been observed in attention disorders such as ADHD^14^ or visual neglect after stroke^15^.

Evidence for these distinct inhibitory and excitatory roles of α- and γ-oscillations in spatial attention mostly stems from imaging studies. Such studies can reveal important relations between neural oscillations and human behavior, but can, however, only provide correlational evidence. In order to establish functional roles of α- and γ-oscillations in this domain, intervention studies modulating the oscillation of interest and monitoring the resulting behavioral changes are required^16^. A method that receives growing popularity to achieve such interventions is transcranial alternating current stimulation (tACS)^17,18^. During tACS, a weak oscillatory current is transcranially applied through the scalp via two or more electrodes. While a large proportion of the current is shunted through the skin, parts of the current reach the underlying cortex where they are thought to synchronize endogenous brain oscillations to the external driving force^17–21^. This way, tACS allows to modulate brain oscillations in a frequency specific manner. Although direct insight into stimulation effects on brain activity in humans are rare due to a massive stimulation artifact introduced to electrophysiological recordings^22^, elevated power in the stimulated frequency band has been repeatedly observed in EEG recordings after stimulation^23–27^. Other studies applied stimulation at frequencies antagonistically coupled to the target oscillation in order to reduce its power. Stimulation in the γ-frequency range, for example, has been shown to attenuate power in the α-frequency band, in line with their antagonistic roles^28,29^.

**Figure 1:**
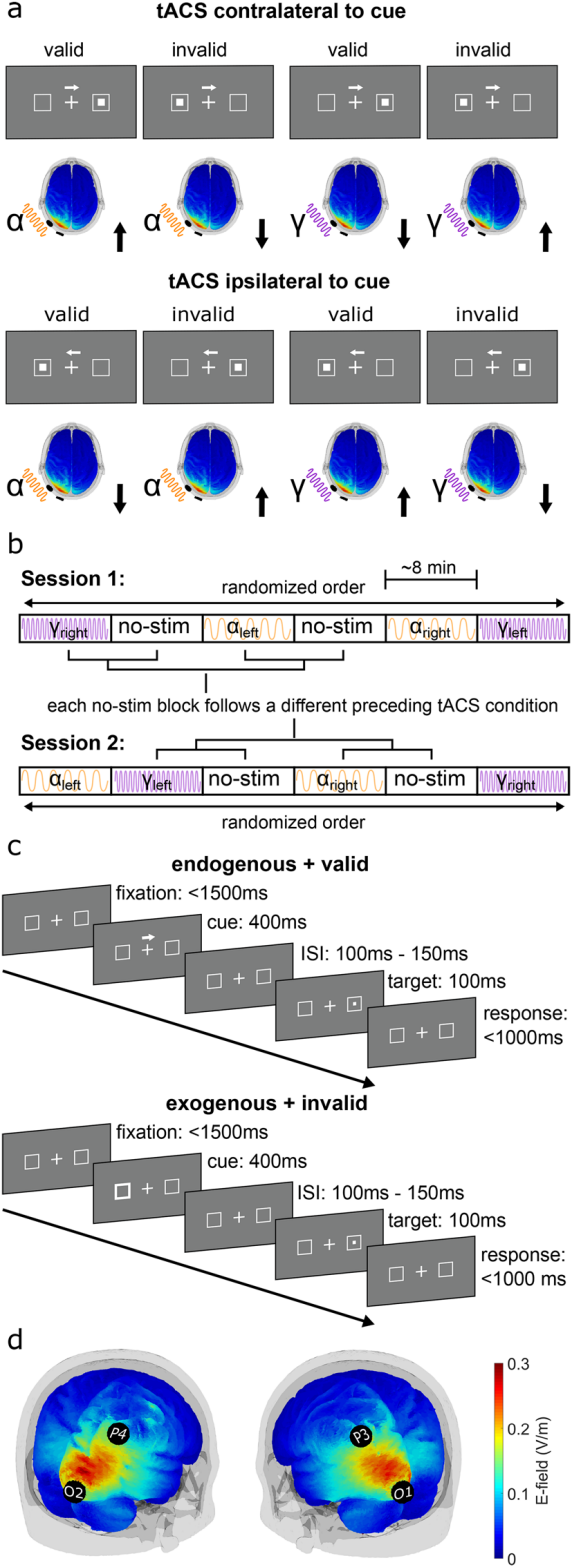
Experimental Design. (**a**) Expected effect of α- versus γ- stimulation on the spatial cueing effect (exemplary for left hemispheric stimulation). Black arrows indicate the expected change in RTs. (**b**) Time-course of the main experiment in two sessions. All four stimulation conditions were applied in one of the six blocks in each session. Blocks were performed in randomized order with the constraint that each of the four stimulation-free blocks (no-stim) follows a different tACS condition. Each of the blocks had a duration of ~ 8-min and contained 50% endogenous and 50% exogenous attention trials. (**c**) Structure of two exemplary trials with an endogenous (top) and an exogenous (bottom) cue. In valid trials (top) the target was presented on the cued location. In invalid trials (bottom) targets were presented opposite to the cued location. ISI: inter stimulus interval. (**d**) Electric field simulation for right and left hemispheric stimulation of the occipital cortex, simulated in an MNI-standard brain. Maps depict the magnitude of the electric field.

Recently, tACS applied in the α-and γ-frequency ranges over left auditory cortex has been shown to differentially modulate endogenous auditory spatial attention^30^. In the current study, we aimed to extent this finding to the visual domain as well as to exogenous attention. To this end, we aimed to increase or decrease interhemispheric α- and γ-power asymmetry by applying lateralized tACS at α- or γ-frequency over the left or right occipital cortex while participants performed a visual-spatial cueing paradigm comprised of endogenous and exogenous cues. We hypothesized that during α-tACS applied to the occipital cortex contralateral to the cued direction, RTs increase after valid cues, while RTs decrease after invalid cues, as compared to RTs after these cues in absence of tACS and during γ-tACS (during γ-tACS contralateral to the cue direction we expected RTs to decrease after valid cues and to increase after invalid cues). We expected the opposite effect when tACS was applied ipsilaterally to the cue direction. Here, we hypothesized that RTs during α-tACS decrease after valid cues, while RTs increase after invalid cues, relative to RTs after these cues in absence of tACS and γ-tACS (during γ-tACS, we expected RTs to increase after valid cues and to decrease after invalid cues; the expected effects are visualized in Fig. 1a).

## Materials and methods

### Participants

A total of 21 participants without reported history of neurological or psychiatric disease volunteered for the study. They all gave written informed consent prior to participation. All were non-smokers, right-handed according to the Edinburgh Handedness Scale^31^ and had normal or corrected to normal vision. On two sessions, approximately one week apart, participants performed a total of 12 blocks of a spatial cueing task while receiving lateralized tACS at individual α- or fixed γ-frequency (47 Hz) or no stimulation over the left or right occipital cortex. Each session was comprised of all experimental conditions, such that the second session was essentially a repetition of the first (except for a different order of stimulation conditions, Fig. 1b) to obtain more data for analysis. One participant aborted the experiment after the first session. Thus, data of 20 participants (25 ± SD: 2.7 years, 10 females), remained for analysis. The study was approved by the ‘Commission for Research Impact assessment and Ethics’ at the University of Oldenburg and performed in accordance with the declaration of Helsinki.

### Experimental design

In each of the two experimental sessions, participants performed six blocks of a spatial cueing paradigm (Fig. 1a, c). Visual stimuli were presented on a computer screen (Samsung P2470H, 1,920 × 1,080 pixels, 60 Hz refresh rate) at a distance of 77 cm. Participants’ head position was maintained using a chin rest. Stimulus presentation was performed using Psychtoolbox 3^32^ running on MATLAB 2016b (The MathWorks Inc, Natick, MA, USA). Participants were instructed to fixate a white cross (0.41° × 0.41° visual angle) on gray background in the center of the screen. Two hollow white squares (each 2.46° × 2.46°) to the left and right of the fixation cross indicated possible locations of the target stimulus. The horizontal distance from the edge of the squares to screen center was 4.42°. Each trial started with a fixation period followed by the presentation of a cue for 400-ms. The cue was either a white arrow, centrally presented above the fixation cross (endogenous cue) pointing towards one of the two target locations, or a brief increase in line-thickness of one of the white squares (exogenous cue). To ensure that participants perceive the cue as informative, a validity of 80% was chosen. A target stimulus (0.12° × 0.12°) was presented in one of the two possible locations 100–150-ms after offset of the cue. The target stimulus was a white rectangle presented in the center of one of the two target locations for a duration of 100-ms (Fig. 1c provides an illustration of the time course of an endogenous and an exogenous attention trial). Participants were instructed to indicate the position of the target stimulus by pressing a button with their left or right index finger. The time window for responses was limited to 1,000-ms after target onset. If participants failed to respond within this window, the trial was counted as incorrect. Manual responses were recorded using custom build response boxes connected to the parallel port of the stimulus PC, allowing to record RTs with sub-millisecond precision. To ensure constant fixation of the central cross, participants’ gaze position was monitored online using an eye-tracker (TheEyeTribe, Copenhagen, Denmark), which sampled at 60 Hz. Gaze data was imported and analyzed online by the MATLAB code controlling the experiment using the EyeTribe Toolbox for MATLAB v0.03 (https://github.com/esdalmaijer/EyeTribe-Toolbox-for-Matlab). A region of interest (ROI) of 4.11° × 4.11° was defined around the central fixation cross. If participants’ gaze position was detected outside the ROI during the time between cue onset and target offset, the trial was aborted. A text was displayed on the screen to inform participants about this. Edges of the ROI reflect a little less than half the distance (46%) between the screen center and the edges of the white squares, which indicate possible target locations. Aborted trials were repeated towards the end of the current block. A total of 180 completed trials (50% endogenous, 50% exogenous) were collected in each block, amounting to a duration of ~ 8 min per block. A nine-point calibration of the eye-tracker was performed in the beginning of each block. Prior to the main experiment, participants were given a practice block to get familiarized with the task and the fixation control.

### Electrical stimulation

During four out of the six blocks in each experimental session, participants received different tACS conditions at α- and γ-frequency delivered to the left and right occipital/parietal cortex. Stimulation was administered in a single-blind design. As stimulation was remote controlled via the MATLAB code that controlled the entire experiment, we decided against a double blinding procedure to allow the experimenter to monitor and ensure the correct and safe tACS application of the system. In each condition, 1 mA (2 mA peak-to-peak) tACS was applied either in the α- (individual α-frequency) or γ- (47 Hz) range and administered to either the left or right occipital cortex. The γ-tACS frequency was chosen to target the center of the lower γ-band (35–65 Hz). During the two remaining blocks no stimulation was applied. All possible stimulation conditions were applied in each experimental session. Contrasting the aforementioned stimulation frequencies and target regions allows to assess the spatial and frequency specificity of tACS effects, as each of these conditions is expected to result in different modulations of participants’ RTs (Fig. 1a). Further this design allows to largely rule out alternative explanations for stimulation effects such as peripheral mechanisms that could give rise to sensory entrainment (e.g. stimulation of the retina or cutaneous nerves)^33–35^. Due to the large distance of the stimulation electrodes to the retina, effects of retinal stimulation (phosphenes) should be spatially unspecific, whereas effects caused by stimulation of cutaneous nerves should cause effects opposite to those expected from a stimulation of the brain, as somatosensory input is processed contralaterally to the stimulated hemisphere. Alternatively, these visual or somatosensory sensations could simply distract participants (without entrainment of oscillations in the brain via sensory pathways). Indeed, after the experimental sessions, 13 participants reported sensations merely at the onset of stimulation, which, however, disappeared after few seconds. Assuming that sensations in the two stimulation conditions cause similar distractions, this would likely give rise to changes in RTs into the same direction for both stimulation frequencies and across cue types. In contrast, for a neurophysiological effect of stimulation we expect a differential effect of the two stimulation frequencies that reverses depending on the validity of the cue*.* Stimulation conditions were performed in randomized order with the constraint that stimulation-free blocks were balanced across the two sessions such that each of the stimulation-free blocks follows one out of the four stimulation conditions (Fig. 1b). Balancing was carried out to avoid systematic influences of carry-over effects from previous tACS conditions^25,26^. A post-hoc inspection indicated a relatively equal distribution of stimulation conditions over time/blocks (Supplementary Fig. S1), ensuring that outlasting effects commonly reported after tACS administration level out across participants. However, as an exception, α-tACS over the right hemisphere was disproportionately often delivered in the first block. TACS was delivered using a hybrid electrical stimulation/EEG system (Starstim 8, Neuroelectrics, Barcelona, Spain), remote controlled using MATLAB 2016a and the MatNic (Neuroelectrics, Barcelona, Spain) interface. Two pairs of circular, sintered Ag/AgCl electrodes (PiStim, Neuroelectrics, Barcelona, Spain) with a radius of 1 cm were placed over locations O1-P3 and O2-P4 of the international 10–20 system, targeting left or right occipital cortex (Fig. 1c). Electrodes were filled with an electrically conductive gel (Signa Gel, Parker Laboratories Inc., Fairfield, NJ, USA). Impedances were kept below 10 kΩ. Stimulation was started with a 1-s fade-in at the beginning of each block of the attention task and maintained until the end of the respective block (~ 8-min). The first trial of each block started during the ramp-in period. Stimulation was faded-out over a 1-s period. The total stimulation duration across blocks was kept below 40-min per day.

Prior to the experiment, participants’ IAF was determined from a 3-min EEG recording during rest with eyes-closed. Signals were recorded at a rate of 250 Hz from electrode Pz (reference: right earlobe, ground: AFz). The EEG signal was filtered between 1 and 40 Hz and segmented into 1-s epochs. Fast Fourier Transforms (2-s zero-padding, Hanning window) were computed on each of the segments and the resulting power spectra were averaged. The power peak in the 8–12 Hz range was used as stimulation frequency for the α-tACS conditions. Due to the much larger frequency band and the lower signal-to-noise ratio, which hinders time efficient estimation of an individual γ-frequency, γ-tACS was applied at a fixed frequency in the center of the lower gamma band (47 Hz). The hybrid electrical stimulation/EEG device allows for time-efficient application of multiple stimulation and EEG electrodes and flexible remote control over stimulation signals. However, due to insufficient dynamic range of the EEG system, the large stimulation artifact resulted in saturation of the EEG signals precluding any analysis of EEG signals during stimulation^22^. We further refrained from analyzing task related EEG due to the low spatial sampling available from the system (4-channels).

### Simulation of electric field

To validate that our stimulation montage targets left and right occipital/parietal areas, respectively, we modelled the to-be expected electric field using the ROAST^36^ toolbox (v2.7). The simulation was performed on a T1-weighted MNI (Montreal Neurological Institute) standard brain. Simulations were carried out separately for the P3-O1 and P4-O2 montages. Electrodes were modelled with a radius of 1 cm, a thickness of 3 mm, a gel layer of 3 mm and a current injection of 1 mA (zero-to-peak). The toolbox simulates the current flow between the pre-defined set of electrodes using a six compartment, finite element model (gray matter, white matter, cerebro-spinal-fluid [csf], bone, skin, air) obtained from a T1-weighted MRI using the SPM12 segmentation algorithm and a post-processing routine to optimize the output for electric field modelling (details on the post-processing can be found in ref. ^36^). Default conductivities of the toolbox were used (WM: 0.126 S/m, GM: 0.276 S/m, csf: 1.65 S/m, bone: 0.01 S/m, skin: 0.465 S/m, air: 2.5e−14 S/m, gel: 0.3 S/m, electrode: 5.9e7 S/m). The resulting electric field maps depicting the magnitude of the field for each electrode montage is shown in Fig. 1d.

### Data analysis

Data analysis was performed using MATLAB 2018a. Statistical analysis was performed using R 3.6.1 (The R Core Team, R Foundation for Statistical Computing, Vienna, Austria) running on R Studio 1.2.5019 (R Studio Team, R Studio Inc. Boston, MA, USA).

Analysis of RTs was carried out exclusively on correct responses. Early responses that were too fast to reflect direct reactions to the onset of the target stimulus, but may rather be attributed to a reaction in response to the cue (< 150-ms after target onset), as well as extremely slow RT (exceeding 4 standard deviations of participants’ individual average RT) were removed from analysis. For statistical analysis, we fitted linear mixed-effects models (LMMs) using the *lme4* package for R. In a first step, we modelled participants’ single trial RTs in stimulation-free blocks as a function of the fixed effects CUEVALIDITY and ATTENTIONTYPE to validate that our task induced a reliable attentional modulation irrespective of the type of attention. We then proceeded to model participants’ single trial RTs as a function of CUEVALIDITY (2 levels, valid vs. invalid), ATTENTIONTYPE (2 levels, endogenous vs. exogenous), CUEDIRECTION (relative to the stimulated hemisphere; 2 levels, ipsi- vs. contralateral), HEMISPHERE (2 levels, left vs. right hemispheric stimulation) and STIMULATION (2 levels, α vs. γ). These factors represent all experimental manipulations carried out in the study and were chosen a priori. To rule out the possibility that the model overfits the data, it was compared to all possible models incorporating subsets of the aforementioned predictors using Akaike’s Information Criterion (AIC). AIC confirmed that this full model was superior in explaining the data as compared to the other possible models (Supplementary Table T1). In all models, a random intercept was allowed for each participant (random effect). LMMs have advantages over traditional Analyses of Variances (ANOVA) as they can take individual variability between participants (e.g. overall tendencies to react faster or slower) into account and leverage single trial data, rather than condition averages. P-values for main effects and interactions were obtained using Satterthwaite approximation implemented in the *anova* function of the *lmerTest* package for R. As there are no standardized measures of effect size available for individual predictors in LMMs, the unstandardized coefficient *b* corresponding to the estimated change in the dependent variable per level of the predictor is reported. Significant interactions were resolved by splitting the data by the levels of one of the predictors. P-values obtained from such follow up analysis were Bonferroni-Holm corrected. In case of significant modulations of RTs by α- versus γ-tACS, conditions were additionally contrasted against RTs of the corresponding experimental conditions in the stimulation-free blocks using the same statistical approach. A full overview of all statistical results of the models is given in Supplementary Tables T2–T11.

## Results

### Spatial cueing effect

**Figure 2:**
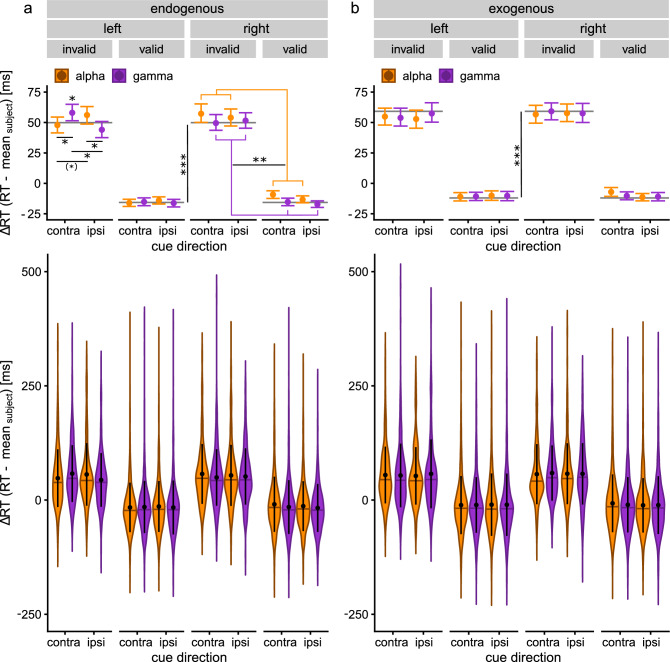
RT differences during α- and γ-tACS over the different experimental conditions. For display purposes RTs are shown after the average RT of each subject over all conditions has been subtracted from single trial data (ΔRTs). Top panels depict condition averages (dots) with their respective 95% confidence intervals (CI, error bars) obtained from non-parametric bootstrapping. Means and CIs were computed from the pooled single trial data of all subjects. Grey, horizontal lines depict average ΔRTs in absence of stimulation. Asterisks indicate significant differences between conditions (^(^*^)^*p* < .1, *p < .05, **p < .01, ***p < .001). Bottom panels show violin plots depicting the range and probability density of single trial ΔRTs across conditions pooled over all subjects underlying the averages in the top panel. Horizontal lines within the violins indicate the median. Black dots depict the mean, vertical black lines indicate the standard deviation (SD). (**a**) ΔRTs during endogenous attention trials. (**b**) ΔRTs during exogenous attention trials. Differences between valid and invalid cues reflect the well documented spatial cueing effect in both attention conditions. While there was no effect of α-/γ-tACS on exogenous attention (**b**), in endogenous attention (**a**), α-tACS over the right hemisphere globally increased RTs compared to γ-tACS and no stimulation, irrespective of the task condition (orange vs. violet bracket, right panel). When applied over the left hemisphere α- and γ-tACS differentially modulated RTs of invalid but not valid endogenous attention trials (left panel).

The linear mixed effects model on single trial RTs in absence of stimulation yielded significant effects of CUEVALIDITY (b = −65.6, F_1,12910_ = 2,357.12, *p* < 0.001) and ATTENTIONTYPE (b = 9.24, F_1,12910_ = 21.05, *p* < 0.001), as well as a significant interaction of the two predictors (b = −5.5, F_1,12910_ = 3.87, *p* = 0.049). Similarly, the linear mixed effects model on single trial RTs during stimulation as a function of CUEVALIDITY, ATTENTIONTYPE, CUEDIRECTION, HEMISPHERE and STIMULATION revealed a strong effect of CUEVALIDITY (LMM, b = -64.1, F_1,26088_ = 4,877.69, *p* < 0.001) and ATTENTIONTYPE (LMM, b = 7.0, F_1,26088.1_ = 19.07, *p* < 0.001). Overall, these results replicate the spatial cueing effect commonly observed in the task. The models predict an RT benefit of ~ 65-ms for valid as compared to invalid cues, indicating that our task successfully induced a spatial cueing effect (Fig. 2a,b). Further, the models suggest slower RTs (~ 7 to 9-ms) during exogenous as compared to endogenous attention trials.

### tACS modulates RTs in endogenous but not exogenous attention

In addition to the two main effects, the LMM on RTs during stimulation yielded significant interactions between STIMULATION, CUEDIRECTION, ATTENTIONTYPE and HEMISPHERE (LMM, b = −36.0, F_1,26088_ = 6.04, *p* = 0.014) and CUETYPE, STIMULATION, CUEDIRECTION, ATTENTIONTYPE and HEMISPHERE (LMM, b = 34.4, F_1,26088_ = 5.07, *p* = 0.024, Supplementary Table T2).

The significant interactions suggest that stimulation may have had different effects on endogenous and exogenous attention. In order to further resolve this relationship, we split the data by the factor ATTENTIONTYPE and fitted two LMMs with predictors CUEVALIDITY, CUEDIRECTION, HEMISPHERE and STIMULATION to separately model RTs of endogenous and exogenous attention trials. The LMM on exogenous trials revealed a strong effect of CUEVALIDITY (LMM, b = −65.8, F_1,12642_ = 2,149.61, *p*_Bonferroni–Holm_ < 0.001), indicative of a spatial cueing effect elicited by exogenous cues. However, no other predictors or interactions turned out significant (all *p* > 0.28, Supplementary Table T3), indicating that tACS did not differentially modulate exogenous spatial attention (Fig. 2b).

Similar to the exogenous attention conditions, RTs during endogenous trials were strongly affected by CUEVALIDITY (LMM, b = −64.1, F_1,13427_ = 2,826.10, *p*_Bonferroni–Holm_ < 0.001), indicative of a spatial cueing effect during endogenous attention trials. However, in addition, this analysis revealed a significant effect of STIMULATION (LMM, b = 9.7, F_1,13427_ = 5.66, *p*_Bonferroni–Holm_ = 0.035) and an interaction between STIMULATION, CUEDIRECTION and HEMISPHERE (LMM, b = 27.0, F_1,13427_ = 10.49, *p*_Bonferroni–Holm_ = 0.002; Fig. 2a; Supplementary Table T4).

### Right occipital α-tACS increases RTs independent of the cue

To further resolve the influence of the stimulated hemisphere on the modulation of endogenous attention, we split the data by the factor HEMISPHERE and subsequently modelled single trial RTs with predictors CUEVALIDITY, CUEDIRECTION and STIMULATION for the two datasets. In line with the previous analyses, we observed strong effects of CUEVALIDITY in both models (right hemisphere: LMM, b = −66.1, F_1,6711_ = 1,424.18, *p*_Bonferroni–Holm_ < 0.001; left hemisphere: LMM, b = −64.0, F_1,6697.1_ = 1,415.01, *p*_Bonferroni–Holm_ < 0.001). For stimulation applied to the right hemisphere, we further observed a significant main effect of STIMULATION (LMM, b = −7.2, F_1,6711.1_ = 7.54, *p* = 0.012). None of the other main effects or interactions were significant (*p* > 0.31, Supplementary Table T5). According to the model slope, RTs were ~ 7-ms shorter during γ- as compared to α-tACS, irrespective of the cued location or cue validity (Fig. 2a, right panel). Additional comparisons against the pooled stimulation-free trials of all blocks indicate that α-tACS increased RTs by ~ 6-ms compared to stimulation-free blocks (LMM, b = 6.0, F_1,9998_ = 10.24, *p* = 0.001, Supplementary Table T6), while there was no difference between γ-tACS and no-stimulation (LMM, b = −0.8, F_1,10016_ < 0.01, *p* = 0.95, Supplementary Table T7). Due to the disproportionally high prevalence of right hemispheric α-tACS in the first block (Supplementary Fig. S1), this result has to be interpreted with caution, as it could have arisen from an order effect. Indeed, when we added the block order as an additional random intercept to the model, the significant difference between α-tACS and no-stimulation vanished (b = −2.8, F_1,3357.3_ = 1.49, *p* = 0.22).

### Differential effects of left occipital α- versus γ-tACS on invalid but not valid trials

Analysis of trials during left hemispheric stimulation yielded significant interactions of STIMULATION and CUEDIRECTION (LMM, b = −22.0, F_1,6697_ = 12.55, *p*_Bonferroni–Holm_ < 0.001) and CUEVALIDITY, STIMULATION and CUEDIRECTION (LMM, b = 18.8, F_1,6697_ = 6.99, *p*_Bonferroni–Holm_ = 0.016; Supplementary Table T8).

As the last interaction indicates an influence of the validity of the cue on the effect of stimulation, we further split endogenous attention trials with left hemispheric stimulation into valid and invalid trials and separately modelled RTs as a function of STIMULATION and CUEDIRECTION. The LMM on valid trials did not reveal any significant main effect or interaction (all *p* > 0.29; Supplementary Table T9), indicating that left hemispheric stimulation did not affect RTs of validly cued trials. In contrast, the LMM on invalidly cued trials, yielded a significant interaction between STIMULATION and CUEDIRECTION (LMM, b = −22.4, F_1,1276.2_ = 11.88, *p*_Bonferroni–Holm_ = 0.001; Fig. 2a, left panel; Supplementary Table T10).

In order to resolve which combinations of stimulation frequency and cue direction (α + ipsi, α + contra, γ + ipsi, γ + contra) drove the interaction, we employed four additional LMMs. Two LMMs were used to contrast RTs after ipsi- versus contralateral cues during α- or γ-tACS, respectively. The other two LMMs were used to contrast RTs during α- versus γ-stimulation after cues ipsi- or contralateral relative to the stimulated hemisphere, respectively. Conceptually, the approach is similar to post-hoc t-tests between factor level combinations in an ANOVA design to resolve the origin of interactions. In addition, we tested if the employed stimulation protocols changed RTs relative to stimulation-free blocks. To this end, we used the pooled invalid endogenous attention trials from all stimulation-free blocks and contrasted these against the aforementioned conditions. P-values obtained from the 8 tests were FDR corrected for multiple comparisons. The analysis yielded significant differences between ipsi- versus contralateral γ-tACS (LMM, b = −14.0, F_1,620.55_ = 9.38, *p*_FDR_ = 0.018), as well as between contralateral α- versus γ-tACS (LMM, b = 10.8, F_1,635.34_ = 5.55, *p*_FDR_ = 0.045) and ipsilateral α- versus γ-tACS (LMM, b = 12.3, F_1,622.36_ = 7.21, *p*_FDR_ = 0.03). Only during contralateral γ-tACS RTs significantly differed compared to no-stimulation (LMM, b = −8.1, F_1,1567.1_, *p*_FDR_ = 0.044; Fig. 2a, top panel, left). The remaining contrasts did not reach significance (Supplementary Table T11).

## Discussion

The current study investigated whether lateralized α- and γ-tACS, aiming to modulate interhemispheric α-/γ-power asymmetry over occipital and posterior-parietal cortices, modulates visual-spatial attention. We found a differential effect of α- and γ-tACS over the left occipital cortex on the spatial cueing effect during endogenous attention. This effect is in line with the excitatory role of γ- and the inhibitory role of α-oscillations^12,13^. The effect was not evident for exogenous attention and appears to be specific to α-/γ-tACS applied over the left occipital cortex. When applied to the right hemisphere, α-tACS appeared to globally increase RTs irrespective of the cue direction or validity. However, due to an imbalance in the order of stimulation conditions this finding has to be interpreted with caution as the effect might be driven by a bias in the order of stimulation conditions. The differential effect of tACS over the left hemisphere on endogenous attention was limited to modulations of RTs in invalid trials, reflecting attentional disengagement and re-orientation.

Our findings are in line with recently reported effects of α- and γ-tACS on endogenous auditory attention^30^. In that study, stimulation of the left auditory cortex resulted in increased performance in response to stimuli presented to the contralateral side during γ-tACS and decreased performance during α-tACS. However, the authors applied tACS exclusively to the left hemisphere, such that the absence of effects in the remaining experimental conditions cannot be compared. Even more recently, α-tACS applied to the left posterior-parietal cortex exclusively modulated endogenous, but not exogenous visual attention or target detection^37^. Again, this pattern of findings is in line with the effect of left-hemispheric α-tACS in our experiment. With the current results we extend previous work, establishing a differential effect of left occipital-parietal α- versus γ-tACS on endogenous but not exogenous visual-spatial attention. Further, our results suggest that effects were driven by modulations of RTs during invalid trials, reflecting attentional disengagement and re-orientation. In another recent study, α- and γ-tACS were applied targeting the right inferior parietal lobe (angular gyrus). Here, α-tACS selectively modulated exogenous attention, while γ-tACS affected endogenous attention^38^.

Interestingly, the differential effect of tACS on endogenous attention in our study was specific to stimulation of the left occipital cortex and endogenous attention. Together these findings may indicate that oscillations in different frequency bands serve distinct roles within different attentional sub-systems. The different effects between hemispheres may further suggest distinct roles of the two hemispheres for controlling α-/γ-power during endogenous attention. While power in the right hemisphere may determine the general level of activation/deactivation, the dynamic in the left hemisphere may predominantly control power asymmetry by up- and down-regulating α- and γ-power relative to the right hemisphere. Indeed, some studies on α-power asymmetry in visual-spatial and auditory attention showed stronger α-power dynamics in the left hemisphere^3,5,6,39^. In one of these studies only α-power modulation in the left hemisphere was predictive of participants attentional performance^3^. In a recent neurofeedback study, Bagherzadeh et al. trained participants to voluntarily control their posterior α-power asymmetry. Irrespective of whether participants had to bias their α-power towards the left or the right hemisphere, they up- or downregulated α-power in the left hemisphere to achieve the required asymmetry, while power in the right hemisphere remained stable^39^. It should be noted, however, that these lateralized differences in α-power dynamics have so far only been reported as unexpected side-findings^3,39^, or are evident from visual inspection of results, but were not explicitly discussed by the authors^5,6^. Thus, more research is needed to systematically evaluate the role of the different hemispheres in α- and γ-power asymmetry. For example, based on the current evidence, it remains elusive if the roles of the hemispheres depend on participants’ handedness (i.e. if left handed individuals may show an opposite effect as reported here). All of the aforementioned studies (and this one) exclusively recorded right-handed participants (except for two left handed volunteers in Ref. ^5^)^3,5,6,39^.

The modulatory effect of α- and γ-tACS in our study was limited to endogenous attention and not observed during exogenous attention. There is ongoing debate if the two types of attention originate from the same brain networks. Most studies suggest the existence of partly overlapping but independent subsystems^1^. These networks differ, among others, with respect to the recruitment of subcortical structures^40–42^, the existence of feedback loops from frontal and parietal areas^43,44^ as well as in their time-courses. For example, P1 and P300 components of the event-related potential exhibit earlier latencies during exogenous as compared to endogenous attention^45^. The differences in the susceptibility to tACS may be seen as additional evidence that endogenous and exogenous attention are represented in distinct subsystems.

In the current study, we applied lateralized α-/γ-tACS with the aim to modulate interhemispheric α-/γ-power asymmetry and observed modulations of RTs during endogenous attention. However, we do not have direct insights to what extent tACS has induced lateralized power changes in the targeted frequency bands due to technical limitations of our EEG system. Assessing effects of tACS on electrophysiological measures imposes a major challenge, as EEG signals are massively contaminated by a strong electromagnetic artifact that cannot be reliably removed up to this point^22^. Given recent successful attempts to uncover online effects of tACS on event-related oscillations^29,46^, future studies may be able to directly observe effects of lateralized α- and γ-tACS on (event-related) inter-hemispheric α-/γ-power asymmetry.

While our results indicate frequency specific effects of α- and γ-tACS on endogenous attention, the role of other frequency bands has not been assessed. We decided to employ no additional control frequencies to keep tACS-dosage and general task demands at a reasonable level for our participants. Future studies should test if similar effects could be achieved by targeting other frequency bands. There is, for example, evidence that oscillations in the β-band show similar power asynchrony patterns over posterior areas as those in the α-band^10^.

It has recently been argued that electric fields inside the brain during conventional tACS might be too weak to modulate endogenous brain oscillations directly^47,48^. Following these concerns, it has been suggested that tACS exerts its effects indirectly via stimulation of peripheral nerves in the retina or the skin^33,34^. This explanation seems, however, incompatible with the distinct spatial pattern of our results. On the one hand, the large distance of stimulation electrodes to the eyes in combination with the partial crossing of visual information in the thalamus, should cause effects of retinal stimulation (i.e. rhythmic phosphenes) to be spatially unspecific. Effects in the current study, however, were limited to stimulation of the left hemisphere. Effects caused by stimulation of cutaneous nerves, on the other hand, should cause effects opposite to those expected from direct stimulation of the brain, as somatosensory input is processed contralaterally to the stimulated hemisphere. This should have given rise to a pattern of stimulation effects opposite to those predicted and observed in the current experiment. Importantly, recent work suggests that tACS can still entrain brain activity even if somatosensory input is blocked^49^ and that the electric field inside the brain, rather than in the periphery, predicts aftereffects of tACS on the power of human brain oscillations^50^. Alternatively, stimulation effects could potentially also arise from distraction of participants by visual or somatosensory sensations (without entrainment of brain oscillations via sensory pathways). Such effects should, however, lead to similar effects (i.e. modulation of RT into the same direction) during α- versus γ-tACS rather than a differential effect as observed in our results. Further it seems unlikely that distraction by sensation causes a specific effect in one experimental condition, rather than a general modulation of RTs across multiple conditions.

Given that psychiatric disorders such as ADHD and visual neglect involve alterations in interhemispheric α-/γ-power asymmetry the current results should be discussed in the light of potential treatment options for such disease. Behavioral effects observed in tACS studies are often relatively small^51^. In the current study, LMMs predict that α- and γ-tACS over the left hemisphere caused changes in RT in the range of ~ 10- to 14-ms. These effects are not sufficiently large to abolish or revert the effect of spatial cueing which was almost sixfold (65-ms) but may be sufficient to bias pathological states of attention in a clinical setting. In recent years different factors have been discussed to contribute to the variability of brain stimulation effects, such as time of day, age, medication and genetic disposition^52^. Further, inter-individual variability in head anatomy and the resulting differences in the induced electric fields may play a major role^50,53–55^. Individually tailored stimulation protocols and montages may substantially reduce variability and elevate stimulation effects in the future. Such protocols should also aim to individualize stimulation frequencies. Mismatches between tACS frequency and the individual, dominant frequency of the target oscillation have repeatedly been suggested as a source of variability^24,50,56^. In the current study, stimulation frequencies were determined from short resting-state recordings. Although this approach provides an estimate of participants IAF that may be closer to the dominant frequency during the task than a fixed frequency, task engagement is known to potentially change the IAF^57,58^. Estimating IAF directly during the task, ideally in a closed-loop setup, may thus further increase tACS effects. In addition, as stimulator systems are relatively cheap, mobile and easy to apply^59,60^, repeated application of tACS e.g. over the course of a week may further enhance stimulation effects. It may thus be worthwhile for future studies to test if patient groups suffering from conditions associated with dysfunctional inter-hemispheric α-/γ-asymmetry could benefit from lateralized α-/γ-tACS.

## Conclusions

The current study aimed to investigate the causal role of α-/γ-asymmetry for endogenous and exogenous visual-spatial attention using tACS. Lateralized α/γ-tACS was able to modulate RTs in the spatial attention task. Noteworthy, the effect was limited to the left hemisphere and endogenous attention. These results provide evidence for distinct roles of α- and γ-oscillations for top-down visual attention and could be indicative of a left hemispheric dominance in the control of α- and γ-power lateralization in the visual cortex.

## Supplementary information

Supplementary file 1 (DOCX 79 kb)

## Data Availability

The datasets generated during and/or analyzed during the current study are available from the corresponding author on reasonable request.

## References

[1] Carrasco M (2011). Visual attention: the past 25 years. Vision Res..

[2] Posner MI, Snyder CR, Davidson BJ (1980). Attention and the detection of signals. J. Exp. Psychol. Gen..

[3] Okazaki YO, De Weerd P, Haegens S, Jensen O (2014). Hemispheric lateralization of posterior alpha reduces distracter interference during face matching. Brain Res..

[4] Haegens S, Handel BF, Jensen O (2011). Top-down controlled alpha band activity in somatosensory areas determines behavioral performance in a discrimination task. J. Neurosci..

[5] Sauseng P (2005). A shift of visual spatial attention is selectively associated with human EEG alpha activity. Eur. J. Neurosci..

[6] Wöstmann M, Herrmann B, Maess B, Obleser J (2016). Spatiotemporal dynamics of auditory attention synchronize with speech. Proc. Natl. Acad. Sci..

[7] Worden MS, Foxe JJ, Wang N, Simpson GV (2000). Anticipatory biasing of visuospatial attention indexed by retinotopically specific alpha-band electroencephalography increases over occipital cortex. J. Neurosci..

[8] Gruber T, Müller MM, Keil A, Elbert T (1999). Selective visual-spatial attention alters induced gamma band responses in the human EEG. Clin. Neurophysiol..

[9] Thut G, Nietzel A, Brandt SA, Pascual-Leone A (2006). Alpha-band electroencephalographic activity over occipital cortex indexes visuospatial attention bias and predicts visual target detection. J. Neurosci..

[10] Siegel M, Donner TH, Oostenveld R, Fries P, Engel AK (2008). Neuronal synchronization along the dorsal visual pathway reflects the focus of spatial attention. Neuron.

[11] Wyart V, Tallon-Baudry C (2008). Neural dissociation between visual awareness and spatial attention. J. Neurosci..

[12] Jensen O, Mazaheri A (2010). Shaping functional architecture by oscillatory alpha activity: gating by inhibition. Front. Hum. Neurosci..

[13] Klimesch W, Sauseng P, Hanslmayr S (2007). EEG alpha oscillations: the inhibition-timing hypothesis. Brain Res. Rev..

[14] ter Huurne N (2013). Behavioral consequences of aberrant alpha lateralization in attention-deficit/hyperactivity disorder. Biol. Psychiatry.

[15] Ros T (2017). Increased alpha-rhythm dynamic range promotes recovery from visuospatial neglect: a neurofeedback study. Neural Plast..

[16] Herrmann CS, Strüber D, Helfrich RF, Engel AK (2016). EEG oscillations: from correlation to causality. Int. J. Psychophysiol..

[17] Herrmann CS, Rach S, Neuling T, Strüber D (2013). Transcranial alternating current stimulation: a review of the underlying mechanisms and modulation of cognitive processes. Front. Hum. Neurosci..

[18] Fröhlich F (2015). Experiments and models of cortical oscillations as a target for noninvasive brain stimulation. Prog. Brain Res..

[19] Fröhlich F, McCormick DA (2010). Endogenous electric fields may guide neocortical network activity. Neuron.

[20] Reato D, Rahman A, Bikson M, Parra LC (2010). Low-intensity electrical stimulation affects network dynamics by modulating population rate and spike timing. J. Neurosci..

[21] Krause MR, Vieira PG, Csorba BA, Pilly PK, Pack CC (2019). Transcranial alternating current stimulation entrains single-neuron activity in the primate brain. Proc. Natl. Acad. Sci..

[22] Kasten FH, Herrmann CS (2019). Recovering brain dynamics during concurrent tACS-M/EEG: an overview of analysis approaches and their methodological and interpretational pitfalls. Brain Topogr..

[23] Zaehle T, Rach S, Herrmann CS (2010). Transcranial alternating current stimulation enhances individual alpha activity in human EEG. PLoS ONE.

[24] Vossen A, Gross J, Thut G (2015). Alpha power increase after transcranial alternating current stimulation at alpha frequency (α-tACS) reflects plastic changes rather than entrainment. Brain Stimul..

[25] Kasten FH, Dowsett J, Herrmann CS (2016). Sustained aftereffect of α-tACS lasts up to 70 min after stimulation. Front. Hum. Neurosci..

[26] Kasten FH, Herrmann CS (2017). Transcranial alternating current stimulation (tACS) enhances mental rotation performance during and after stimulation. Front. Hum. Neurosci..

[27] Wischnewski M (2018). NMDA receptor-mediated motor cortex plasticity after 20 Hz transcranial alternating current stimulation. Cereb. Cortex.

[28] Boyle, M. R. & Frohlich, F. EEG feedback-controlled transcranial alternating current stimulation. in *2013 6th International IEEE/EMBS Conference on Neural Engineering (NER)* 140–143 (IEEE, 2013). 10.1109/NER.2013.6695891.

[29] Herring JD, Esterer S, Marshall TR, Jensen O, Bergmann TO (2019). Low-frequency alternating current stimulation rhythmically suppresses gamma-band oscillations and impairs perceptual performance. Neuroimage.

[30] Wöstmann M, Vosskuhl J, Obleser J, Herrmann CS (2018). Opposite effects of lateralised transcranial alpha versus gamma stimulation on auditory spatial attention. Brain Stimul..

[31] Oldfield RCC (1971). The assessment and analysis of handedness: the Edinburgh inventory. Neuropsychologia.

[32] Kleiner, M., Brainard, D. & Pelli, D. What’s new in Psychtoolbox-3? in *Perception 36 ECVP Abstract Supplement* (2007). 10.1068/v070821.

[33] Asamoah B, Khatoun A, Mc Laughlin M (2019). tACS motor system effects can be caused by transcutaneous stimulation of peripheral nerves. Nat. Commun..

[34] Schutter DJLG (2016). Cutaneous retinal activation and neural entrainment in transcranial alternating current stimulation: a systematic review. Neuroimage.

[35] Kar K, Krekelberg B (2012). Transcranial electrical stimulation over visual cortex evokes phosphenes with a retinal origin. J. Neurophysiol..

[36] Huang Y, Datta A, Bikson M, Parra LC (2019). Realistic volumetric-approach to simulate transcranial electric stimulation—ROAST—a fully automated open-source pipeline. J. Neural Eng..

[37] Schuhmann T (2019). Left parietal tACS at alpha frequency induces a shift of visuospatial attention. PLoS ONE.

[38] Hopfinger JB, Parsons J, Fröhlich F (2017). Differential effects of 10-Hz and 40-Hz transcranial alternating current stimulation (tACS) on endogenous versus exogenous attention. Cogn. Neurosci..

[39] Bagherzadeh Y, Baldauf D, Pantazis D, Desimone R (2020). Alpha synchrony and the neurofeedback control of spatial attention. Neuron.

[40] Robinson DL, Bowman EM, Kertzman C (1995). Covert orienting of attention in macaques. II. Contributions of parietal cortex. J. Neurophysiol..

[41] Zackon DH, Casson EJ, Zafar A, Stelmach L, Racette L (1999). The temporal order judgment paradigm: subcorticalattentional contribution under exogenous and endogenouscueing conditions. Neuropsychologia.

[42] McAlonan K, Cavanaugh J, Wurtz RH (2008). Guarding the gateway to cortex with attention in visual thalamus. Nature.

[43] Bressler SL, Tang W, Sylvester CM, Shulman GL, Corbetta M (2008). Top-down control of human visual cortex by frontal and parietal cortex in anticipatory visual spatial attention. J. Neurosci..

[44] Buffalo EA, Fries P, Landman R, Liang H, Desimone R (2010). A backward progression of attentional effects in the ventral stream. Proc. Natl. Acad. Sci..

[45] Hopfinger JB, West VM (2006). Interactions between endogenous and exogenous attention on cortical visual processing. Neuroimage.

[46] Kasten FH, Maess B, Herrmann CS (2018). Facilitated event-related power modulations during transcranial alternating current stimulation (tACS) revealed by concurrent tACS-MEG. eNeuro.

[47] Lafon B (2017). Low frequency transcranial electrical stimulation does not entrain sleep rhythms measured by human intracranial recordings. Nat. Commun..

[48] Vöröslakos M (2018). Direct effects of transcranial electric stimulation on brain circuits in rats and humans. Nat. Commun..

[49] Vieira, P. G., Krause, M. R. & Pack, C. C. tACS entrains neural activity while somatosensory input is blocked. *bioRxiv* 691022 (2019). 10.1101/691022. 10.1371/journal.pbio.3000834 PMC7553316 33001971

[50] Kasten FH, Duecker K, Maack MC, Meiser A, Herrmann CS (2019). Integrating electric field modeling and neuroimaging to explain inter-individual variability of tACS effects. Nat. Commun..

[51] Schutter DJLG, Wischnewski M (2016). A meta-analytic study of exogenous oscillatory electric potentials in neuroenhancement. Neuropsychologia.

[52] Ridding MC, Ziemann U (2010). Determinants of the induction of cortical plasticity by non-invasive brain stimulation in healthy subjects. J. Physiol..

[53] Laakso I, Tanaka S, Koyama S, De Santis V, Hirata A (2015). Inter-subject variability in electric fields of motor cortical tDCS. Brain Stimul..

[54] Antonenko D (2019). Towards precise brain stimulation: is electric field simulation related to neuromodulation?. Brain Stimul..

[55] Laakso I, Mikkonen M, Koyama S, Hirata A, Tanaka S (2019). Can electric fields explain inter-individual variability in transcranial direct current stimulation of the motor cortex?. Sci. Rep..

[56] Stecher HI, Herrmann CS (2018). Absence of alpha-tACS aftereffects in darkness reveals importance of taking derivations of stimulation frequency and individual alpha variability into account. Front. Psychol..

[57] Haegens S, Cousijn H, Wallis G, Harrison PJ, Nobre AC (2014). Inter- and intra-individual variability in alpha peak frequency. Neuroimage.

[58] Benwell CSY (2019). Frequency and power of human alpha oscillations drift systematically with time-on-task. Neuroimage.

[59] Antal A (2017). Low intensity transcranial electric stimulation: safety, ethical, legal regulatory and application guidelines. Clin. Neurophysiol..

[60] Bikson M (2018). Rigor and reproducibility in research with transcranial electrical stimulation: an NIMH-sponsored workshop. Brain Stimul..

