# Supplementary material for: Hemisphere-specific, differential effects of lateralized, occipital–parietal α- versus γ-tACS on endogenous but not exogenous visual-spatial attention
Source: Sci Rep. 2020 Jul 23;10:12270. doi: 10.1038/s41598-020-68992-2 (PMC7378174; doi:10.1038/s41598-020-68992-2)
Supplement: Supplementary file 1 — (DOCX 79 kb) [file 41598_2020_68992_MOESM1_ESM.docx]

Supplementary Information

Hemisphere-specific, differential effects of lateralized, occipital-parietal α- vs. γ-tACS on endogenous but not exogenous visual-spatial attention

Florian H. Kasten^1,2^, Tea Wendeln^1^, Heiko I. Stecher^1^, and Christoph S. Herrmann^1,2,3,*^

^1^Experimental Psychology Lab, Department of Psychology, Cluster for Excellence “Hearing for All”, European Medical School, Carl von Ossietzky University, Oldenburg, Germany

^2^Neuroimaging Unit, European Medical School, Carl von Ossietzky University, Oldenburg, Germany

^3^Research Center Neurosensory Science, Carl von Ossietzky University Oldenburg, Germany

*****Corresponding author. Experimental Psychology Lab, Carl von Ossietzky University, Oldenburg, Ammerlaender Heerstr. 114-118, 26129 Oldenburg, Germany. E-mail address: *christoph.herrmann@uni-oldenburg.de*, phone: +*49 441 798 4936*

**Supplementary Table T1:** Akaike’s Information Criterion (AIC) for all possible LMM variants.

| **Model** | **AIC** |
| --- | --- |
| Random Intercept Model | 293715.2 |
| CUEVALIDITY | 289241.5 |
| STIMULATION | 293711.5 |
| CUEDIRECTION | 293714.0 |
| ATTENTIONTYPE | 293689.1 |
| HEMISPHERE | 293713.1 |
| CUEVALIDITY x STIMULATION | 289236.5 |
| CUEVALIDITY x CUEDIRECTION | 289239.3 |
| STIMULATION x CUEDIRECTION | 293709.5 |
| CUEVALIDITY x ATTENTIONTYPE | 289208.9 |
| STIMULATION x ATTENTIONTYPE | 293681.6 |
| CUEDIRECTION x ATTENTIONTYPE | 293686.8 |
| CUEVALIDITY x HEMISPHERE | 289237.7 |
| STIMULATION x HEMISPHERE | 293705.9 |
| CUEDIRECTION x HEMISPHERE | 293709.2 |
| ATTENTIONTYPE x HEMISPHERE | 293686.0 |
| CUEVALIDITY x STIMULATION x CUEDIRECTION | 289229.9 |
| CUEVALIDITY x STIMULATION x ATTENTIONTYPE | 289197.3 |
| CUEVALIDITY x CUEDIRECTION x ATTENTIONTYPE | 289203.2 |
| STIMULATION x CUEDIRECTION x ATTENTIONTYPE | 293674.5 |
| CUEVALIDITY x STIMULATION x HEMISPHERE | 289226.0 |
| CUEVALIDITY x CUEDIRECTION x HEMISPHERE | 289228.4 |
| STIMULATION x CUEDIRECTION x HEMISPHERE | 293695.9 |
| CUEVALIDITY x ATTENTIONTYPE x HEMISPHERE | 289201.3 |
| STIMULATION x ATTENTIONTYPE x HEMISPHERE | 293672.0 |
| CUEDIRECTION x ATTENTIONTYPE x HEMISPHERE | 293678.7 |
| CUEVALIDITY x STIMULATION x CUEDIRECTION x ATTENTIONTYPE | 289178.7 |
| CUEVALIDITY x STIMULATION x CUEDIRECTION x HEMISPHERE | 289202.3 |
| CUEVALIDITY x STIMULATION x ATTENTIONTYPE x HEMISPHERE | 289176.1 |
| CUEVALIDITY x CUEDIRECTION x ATTENTIONTYPE x HEMISPHERE | 289182.3 |
| STIMULATION x CUEDIRECTION x ATTENTIONTYPE x HEMISPHERE | 293649.8 |
| **CUEVALIDITY x STIMULATION x CUEDIRECTION x ATTENTIONTYPE x HEMISPHERE** | **289118.5** |

*Note.* Each row represents the AIC for a candidate LMM consisting of a subset of predictors which reflect experimental manipulations carried out in the experiment. In all models, a random intercept was allowed for each subject. Models were fitted on the whole dataset. The model with the lowest AIC is indicated in bold font.

**Supplementary Table T2:** Results of LMM on single trial RT

| **Predictor** | ***b*** | ***df_Num_*** | ***df_Den_*** | ***F*** | ***p*** |
| --- | --- | --- | --- | --- | --- |
| CUEVALIDITY | -64.1 | 1 | 26088 | 4877.69 | < .001* |
| STIMULATION | 10.1 | 1 | 26088.1 | 1.78 | .18 |
| CUEDIRECTION | 8.3 | 1 | 26088 | 0.86 | .35 |
| ATTENTIONTYPE | 7.0 | 1 | 26088.1 | 19.07 | < .001* |
| HEMISPHERE | 9.3 | 1 | 26088 | 3.16 | .08 |
| CUEVALIDITY x STIMULATION | -9.1 | 1 | 26088 | 0.25 | .62 |
| CUEVALIDITY x CUEDIRECTION | -6.2 | 1 | 26088 | 0.04 | .85 |
| STIMULATION x CUEDIRECTION | -22.2 | 1 | 26088 | 0.56 | .45 |
| CUEVALIDITY x ATTENTIONTYPE | -1.9 | 1 | 26088 | 0.04 | .85 |
| STIMULATION x ATTENTIONTYPE | -11.3 | 1 | 26088 | 3.06 | .08 |
| CUEDIRECTION x ATTENTIONTYPE | -10.3 | 1 | 26088 | 0.36 | .55 |
| CUEVALIDITY x HEMISPHERE | -2.4 | 1 | 26088 | 0.37 | .54 |
| STIMULATION x HEMISPHERE | -17.8 | 1 | 26088 | 1.80 | .18 |
| CUEDIRECTION x HEMISPHERE | -11.4 | 1 | 26088 | 0.54 | .46 |
| ATTENTIONTYPE x HEMISPHERE | -7.5 | 1 | 26088 | 0.01 | .92 |
| CUEVALIDITY x STIMULATION x CUEDIRECTION | 19.0 | 1 | 26088 | 1.10 | .29 |
| CUEVALIDITY x STIMULATION x ATTENTIONTYPE | 10.5 | 1 | 26088 | 0.39 | .53 |
| CUEVALIDITY x CUEDIRECTION x ATTENTIONTYPE | 9.1 | 1 | 26088 | 0.18 | .67 |
| STIMULATION x CUEDIRECTION x ATTENTIONTYPE | 28.1 | 1 | 26088 | 2.55 | .11 |
| CUEVALIDITY x STIMULATION x HEMISPHERE | 10.7 | 1 | 26088 | 0.02 | .88 |
| CUEVALIDITY x CUEDIRECTION x HEMISPHERE | 5.2 | 1 | 26088 | 1.02 | .31 |
| STIMULATION x CUEDIRECTION x HEMISPHERE | 27.5 | 1 | 26088 | 3.41 | .07 |
| CUEVALIDITY x ATTENTIONTYPE x HEMISPHERE | 4.4 | 1 | 26088 | 0.45 | .50 |
| STIMULATION x ATTENTIONTYPE x HEMISPHERE | 21.5 | 1 | 26088 | 0.71 | .40 |
| CUEDIRECTION x ATTENTIONTYPE x HEMISPHERE | 14.5 | 1 | 26088 | 0.18 | .67 |
| CUEVALIDITY x STIMULATION x CUEDIRECTION x ATTENTIONTYPE | -25.2 | 1 | 26088 | 1.11 | .29 |
| CUEVALIDITY x STIMULATION x CUEDIRECTION x HEMISPHERE | -22.0 | 1 | 26088 | 0.39 | .53 |
| CUEVALIDITY x STIMULATION x ATTENTIONTYPE x HEMISPHERE | -17.8 | 1 | 26088 | 0.01 | .94 |
| CUEVALIDITY x CUEDIRECTION x ATTENTIONTYPE x HEMISPHERE | -13.4 | 1 | 26088 | 0.25 | .62 |
| STIMULATION x CUEDIRECTION x ATTENTIONTYPE x HEMISPHERE | -36 | 1 | 26088 | 6.04 | 0.014* |
| CUEVALIDITY x STIMULATION x CUEDIRECTION x ATTENTIONTYPE x HEMISPHERE | 34.4 | 1 | 26088 | 5.07 | 0.024* |

*Note*. *df_Num_* indicates degrees of freedom numerator. *df_Den_* indicated degrees of freedom denominator. The unstandardized coefficient b represents the amount of change in the response variable per change in the level of the predictor. Asterisks indicate significance (* p < .05).

**Supplementary Table T3:** Results of LMM on single trial RT during exogenous attention

| **Predictor** | ***b*** | ***df_Num_*** | ***df_Den_*** | ***F*** | ***p*** |
| --- | --- | --- | --- | --- | --- |
| CUEVALIDITY | -65.8 | 1 | 12642 | 2149.61 | < .001* |
| STIMULATION | -1.1 | 1 | 12642.1 | 0.09 | .76 |
| CUEDIRECTION | -2.0 | 1 | 12642.1 | 0.04 | .84 |
| HEMISPHERE | 1.9 | 1 | 12642 | 1.76 | .36 |
| CUEVALIDITY x STIMULATION | 1.3 | 1 | 12642 | 0.57 | .90 |
| CUEVALIDITY x CUEDIRECTION | 2.9 | 1 | 12642.1 | 0.19 | 1.0 |
| STIMULATION x CUEDIRECTION | 5.7 | 1 | 12642 | 0.32 | .57 |
| CUEVALIDITY x HEMISPHERE | 2.1 | 1 | 12642 | 0.81 | .74 |
| STIMULATION x HEMISPHERE | 3.6 | 1 | 12642 | 0.12 | .73 |
| CUEDIRECTION x HEMISPHERE | 3.0 | 1 | 12642.1 | 0.61 | .87 |
| CUEVALIDITY x STIMULATION x CUEDIRECTION | -6.1 | 1 | 12642 | < 0.01 | .98 |
| CUEVALIDITY x STIMULATION x HEMISPHERE | -7.1 | 1 | 12642 | 0.02 | 1.0 |
| CUEVALIDITY x CUEDIRECTION x HEMISPHERE | -8.4 | 1 | 12642 | 0.15 | .70 |
| STIMULATION x CUEDIRECTION x HEMISPHERE | -8.4 | 1 | 12642 | 0.14 | .71 |
| CUEVALIDITY x STIMULATION x CUEDIRECTION x HEMISPHERE | 12.4 | 1 | 12642 | 1.17 | .28 |

*Note*. *df_Num_* indicates degrees of freedom numerator. *df_Den_* indicated degrees of freedom denominator. The unstandardized coefficient *b* represents the amount of change in the response variable per change in the level of the predictor. Asterisks code for significance Asterisks indicate significance (* p < .05). P-values are Bonferroni-Holm corrected for two multiple comparisons with the LMM results on the other half of the data (Supplementary Table T3).

**Supplementary Table T4:** Results of LMM on single trial RT during endogenous attention

| **Predictor** | ***b*** | ***df_Num_*** | ***df_Den_*** | ***F*** | ***p*** |
| --- | --- | --- | --- | --- | --- |
| CUEVALIDITY | -64.1 | 1 | 13427 | 2826.10 | < .001* |
| STIMULATION | 9.7 | 1 | 13427 | 5.66 | 0.035* |
| CUEDIRECTION | 8.1 | 1 | 13427 | 1.30 | 0.51 |
| HEMISPHERE | 9.1 | 1 | 13427 | 1.81 | 0.36 |
| CUEVALIDITY x STIMULATION | -8.8 | 1 | 13427 | 0.01 | 0.93 |
| CUEVALIDITY x CUEDIRECTION | -6.0 | 1 | 13427 | 0.02 | 1.0 |
| STIMULATION x CUEDIRECTION | -21.9 | 1 | 13427 | 3.11 | 0.15 |
| CUEVALIDITY x HEMISPHERE | -2.1 | 1 | 13427 | < 0.01 | 0.99 |
| STIMULATION x HEMISPHERE | -17.2 | 1 | 13427 | 2.52 | 0.23 |
| CUEDIRECTION x HEMISPHERE | -11.1 | 1 | 13427 | 0.04 | 0.87 |
| CUEVALIDITY x STIMULATION x CUEDIRECTION | 18.7 | 1 | 13427 | 2.51 | 0.23 |
| CUEVALIDITY x STIMULATION x HEMISPHERE | 10.1 | 1 | 13427 | 0.01 | 1.0 |
| CUEVALIDITY x CUEDIRECTION x HEMISPHERE | 4.8 | 1 | 13427 | 1.37 | .48 |
| STIMULATION x CUEDIRECTION x HEMISPHERE | 27.0 | 1 | 13427 | 10.49 | .002* |
| CUEVALIDITY x STIMULATION x CUEDIRECTION x HEMISPHERE | -21.4 | 1 | 13427 | 4.54 | .067 |

*Note*. *df_Num_* indicates degrees of freedom numerator. *df_Den_* indicated degrees of freedom denominator. The unstandardized coefficient *b* represents the amount of change in the response variable per change in the level of the predictor. Asterisks indicate significance (* p < .05). P-values are Bonferroni-Holm corrected for two multiple comparisons with the LMM results on the other half of the data (Supplementary Table T2).

**Supplementary Table T5:** Results of LMM on RT during endogenous attention with tACS applied to the right hemisphere

| **Predictor** | ***b*** | ***df_Num_*** | ***df_Den_*** | ***F*** | ***p*** |
| --- | --- | --- | --- | --- | --- |
| CUEVALIDITY | -66.1 | 1 | 6711 | 1424.18 | < .001* |
| STIMULATION | -7.2 | 1 | 6711.1 | 7.54 | .012* |
| CUEDIRECTION | -3.0 | 1 | 6711 | 0.97 | .64 |
| CUEVALIDITY x STIMULATION | 1.1 | 1 | 6711 | < 0.00 | 1.0 |
| CUEVALIDITY x CUEDIRECTION | -1.2 | 1 | 6711 | 0.49 | .71 |
| STIMULATION x CUEDIRECTION | 4.9 | 1 | 6711 | 1.06 | .31 |
| CUEVALIDITY x STIMULATION x CUEDIRECTION | -2.5 | 1 | 6711 | 0.13 | .72 |

*Note*. *df_Num_* indicates degrees of freedom numerator. *df_Den_* indicated degrees of freedom denominator. The unstandardized coefficient *b* represents the amount of change in the response variable per change in the level of the predictor. Asterisks indicate significance (* p < .05). P-values are Bonferroni-Holm corrected for two multiple comparisons with the LMM results on the other half of the data (Supplementary Table T7).

**Supplementary Table T6:** Results of LMM on RT during endogenous attention with right hemispheric alpha tACS vs. RTs in matched trials (endogenous attention) during stimulation free blocks.

| **Predictor** | ***b*** | ***df_Num_*** | ***df_Den_*** | ***F*** | ***p*** |
| --- | --- | --- | --- | --- | --- |
| CUEVALIDITY | -67.0 | 1 | 9998 | 1758.93 | < .001* |
| STIMULATION | -6.0 | 1 | 9998 | 10.24 | < .001* |
| CUEVALIDITY x STIMULATION | 1.0 | 1 | 9998 | 0.15 | .70 |

*Note*. *df_Num_* indicates degrees of freedom numerator. *df_Den_* indicated degrees of freedom denominator. The unstandardized coefficient *b* represents the amount of change in the response variable per change in the level of the predictor. Asterisks indicate significance (* p < .05).

**Supplementary Table T7:** Results of LMM on RT during endogenous attention with right hemispheric gamma tACS vs. RTs in matched trials (endogenous attention) during stimulation free blocks.

| **Predictor** | ***b*** | ***df_Num_*** | ***df_Den_*** | ***F*** | ***p*** |
| --- | --- | --- | --- | --- | --- |
| CUEVALIDITY | -66.9 | 1 | 10016 | 1814.46 | < .001* |
| STIMULATION | -0.8 | 1 | 10016.1 | < 0.01 | .95 |
| CUEVALIDITY x STIMULATION | 1.3 | 1 | 10016 | 0.18 | .67 |

*Note*. *df_Num_* indicates degrees of freedom numerator. *df_Den_* indicated degrees of freedom denominator. The unstandardized coefficient *b* represents the amount of change in the response variable per change in the level of the predictor. Asterisks indicate significance (* p < .05).

**Supplementary Table T8:** Results of LMM on RT during endogenous attention with tACS applied to the left hemisphere.

| **Predictor** | ***b*** | ***df_Num_*** | ***df_Den_*** | ***F*** | ***p*** |
| --- | --- | --- | --- | --- | --- |
| CUEVALIDITY | -64.0 | 1 | 6697.1 | 1415.01 | < .001* |
| STIMULATION | 9.8 | 1 | 6697.1 | 0.31 | .58 |
| CUEDIRECTION | 8.2 | 1 | 6697 | 0.44 | .65 |
| CUEVALIDITY x STIMULATION | -8.9 | 1 | 6697 | 0.02 | 1.0 |
| CUEVALIDITY x CUEDIRECTION | -6.1 | 1 | 6697 | 0.86 | .71 |
| STIMULATION x CUEDIRECTION | -22.0 | 1 | 6697 | 12.55 | < .001* |
| CUEVALIDITY x STIMULATION x CUEDIRECTION | 18.8 | 1 | 6697 | 6.99 | .016* |

*Note*. *df_Num_* indicates degrees of freedom numerator. *df_Den_* indicated degrees of freedom denominator. The unstandardized coefficient *b* represents the amount of change in the response variable per change in the level of the predictor. Asterisks indicate significance (* p < .05). P-values are Bonferroni-Holm corrected for two multiple comparisons with the LMM results on the other half of the data (Supplementary Table T4).

**Supplementary Table T9:** Results of LMM on RT in valid trials during endogenous attention with tACS applied to the left hemisphere.

| **Predictor** | ***b*** | ***df_Num_*** | ***df_Den_*** | ***F*** | ***p*** |
| --- | --- | --- | --- | --- | --- |
| STIMULATION | 0.8 | 1 | 5402 | 0.26 | 1.0 |
| CUEDIRECTION | 2.0 | 1 | 5402 | 0.08 | .91 |
| STIMULATION x CUEDIRECTION | -3.2 | 1 | 5402 | 1.11 | .29 |

*Note*. *df_Num_* indicates degrees of freedom numerator. *df_Den_* indicated degrees of freedom denominator. The unstandardized coefficient *b* represents the amount of change in the response variable per change in the level of the predictor. Asterisks indicate significance (* p < .05). P-values are Bonferroni-Holm corrected for two multiple comparisons with the LMM results on the other half of the data (Supplementary Table T9).

**Supplementary Table T10:** Results of LMM on RT in invalid trials during endogenous attention with tACS applied to the left hemisphere.

| **Predictor** | ***b*** | ***df_Num_*** | ***df_Den_*** | ***F*** | ***p*** |
| --- | --- | --- | --- | --- | --- |
| STIMULATION | 10.5 | 1 | 1276.2 | 0.04 | 1.0 |
| CUEDIRECTION | 8.8 | 1 | 1276.2 | 0.55 | .92 |
| STIMULATION x CUEDIRECTION | -22.4 | 1 | 1276.2 | 11.88 | .0012* |

*Note*. *df_Num_* indicates degrees of freedom numerator. *df_Den_* indicated degrees of freedom denominator. The unstandardized coefficient *b* represents the amount of change in the response variable per change in the level of the predictor. Asterisks indicate significance (* p < .05). P-values are Bonferroni-Holm corrected for two multiple comparisons with the LMM results on the other half of the data (Supplementary Table T8).

**Supplementary Table T11:** Post-hoc LMMs. left hemispheric tACS invalid trials

| **Predictor** | ***b*** | ***df_Num_*** | ***df_Den_*** | ***F*** | ***p_FDR_*** |
| --- | --- | --- | --- | --- | --- |
| CUEVALIDITY (alpha) | 8.9 | 1 | 637.16 | 3.74 | .086^(^*^)^ |
| CUEVALIDITY (gamma) | -14.0 | 1 | 620.55 | 9.38 | .018* |
| STUMULATION (ipsi) | -12.3 | 1 | 622.36 | 7.21 | .030* |
| STIMULATION (contra) | 10.8 | 1 | 635.4 | 5.55 | .045* |
| STIMULATION (alpha ipsi vs. no stim) | -6.22 | 1 | 1569.1 | 3.08 | .11 |
| STIMULATION (alpha contra vs. no stim) | 2.3 | 1 | 1573.1 | 0.43 | .51 |
| STIMULATION (gamma ipsi vs. no stim) | 5.9 | 1 | 1558.2 | 2.77 | .11 |
| STIMULATION (gamma contra vs. no stim) | -8.1 | 1 | 1567.1 | 5.23 | .044* |

*Note*. *df_Num_* indicates degrees of freedom numerator. *df_Den_* indicated degrees of freedom denominator. The unstandardized coefficient *b* represents the amount of change in the response variable per change in the level of the predictor. Asterisks indicate significance ((*) p < .1, * p < .05).

**
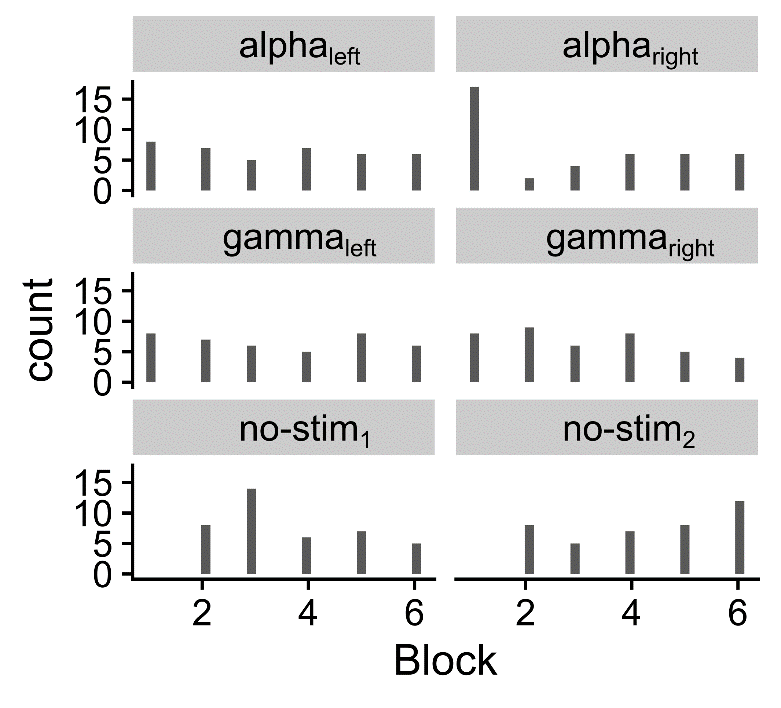
Supplementary Figure S1:** Distribution of stimulation conditions over time (blocks). Bars represent the number of times a stimulation condition was performed in a particular block (across all sessions and all participants). Please note that due to the counterbalancing, stimulation free blocks (no-stim) were never occured as the first block.
